# Supplementary material for: The Systems Biology Research Tool: evolvable open-source software
Source: BMC Syst Biol. 2008 Jun 29;2:55. doi: 10.1186/1752-0509-2-55 (PMC2446383; doi:10.1186/1752-0509-2-55)
Supplement: Additional file 1 — SBRT Archive. An archive of the current version of the Systems Biology Research Tool. [file 1752-0509-2-55-S1.zip › sbrt-1.4.0/doc/developers_guide/api/sbrt/kernel/fba/ReversibleRxn.html]

ReversibleRxn


|  |  |  |
| --- | --- | --- |
| **Overview** \| **Package** \| **Class** \| **Use** \| **Tree** \| **Deprecated** \| **Index** \| **Help** | |  |
| **PREV CLASS**   **NEXT CLASS** | **FRAMES**    **NO FRAMES**     **All Classes** |  |
| SUMMARY: NESTED \| FIELD \| CONSTR \| METHOD | DETAIL: FIELD \| CONSTR \| METHOD |  |


---


## sbrt.kernel.fba Class ReversibleRxn

```
java.lang.Object
  sbrt.kernel.fba.AbstractRxn
      sbrt.kernel.fba.ReversibleRxn
```

**All Implemented Interfaces:**: ChemRxn

---

``` public class ReversibleRxn extends AbstractRxn ```

This class is used to represent reversible chemical reactions.

Instances of this class are immutable.

**Author:**
:   This class was written and documented by Jeremiah Wright while in the
    Wagner Lab.

---

**Constructor Summary**

`ReversibleRxn(LinearComb<java.lang.String> reactants,
LinearComb<java.lang.String> products)`
  
          Constructs a new reversible reaction from the provided reactants and
products.


| **Method Summary** | |
| --- | --- |
| `boolean` | `isEquivalent(ChemRxn rxn)`              Indicates if the provided chemical reaction is stoichiometrically equivalent to this reaction. |
| `boolean` | `isReverse(ChemRxn rxn)`             Returns `false`, since no reaction can be the "reverse" of a reversible reaction. |
| `java.lang.String` | `toString()`             Returns an appropriate string representation of this reversible reaction. |

**Methods inherited from class sbrt.kernel.fba.AbstractRxn**

`equals, getProducts, getRatio, getReactants, hashCode, main`

**Methods inherited from class java.lang.Object**

`clone, finalize, getClass, notify, notifyAll, wait, wait, wait`

**Constructor Detail**

### ReversibleRxn

```
public ReversibleRxn(LinearComb<java.lang.String> reactants,
                     LinearComb<java.lang.String> products)
```

:   Constructs a new reversible reaction from the provided reactants and
    products.

    **Parameters:**: `reactants` - the linear combination of chemical species representing the "left-hand side" of the reaction.: `products` - the linear combination of chemical species representing the "right-hand side" of the reaction. **Throws:**: `java.lang.NullPointerException` - if the reactants or products are `null`, or if any chemical species within them is `null`.: `java.lang.IllegalArgumentException` - if • the reactants or products contain a common chemical species, • either linear combination contains a non-zero constant, • a stoichiometric coefficient is NaN, infinite, or ≤ 0.


**Method Detail**

### isEquivalent

```
public boolean isEquivalent(ChemRxn rxn)
```

:   Indicates if the provided chemical reaction is stoichiometrically
    equivalent to this reaction.

    :   **Parameters:**: `rxn` - the reaction to test for equivalence. **Returns:**: `true` if both reactions are equivalent; `false` otherwise.

---


### isReverse

```
public boolean isReverse(ChemRxn rxn)
```

:   Returns `false`, since no reaction can be the "reverse" of a
    reversible reaction. The concept of a reverse reaction is not meaningful
    for reversible reactions. When referring to the "forward" and "reverse"
    reactions of a reversible reaction, the reaction has been implicity
    decomposed into two irreversible reactions; and the labels "forward" and
    "reverse" are relative to the way in which the reaction is written, which
    is arbitrary.
    `isEquivalent(ChemRxn)` is a more meaningful comparison for
    reversible reactions.

    :   **Parameters:**: `rxn` - the reaction to test. **Returns:**: `false` **See Also:**: `IrrevRxn`

---


### toString

```
public java.lang.String toString()
```

:   Returns an appropriate string representation of this reversible reaction.

    :   **Specified by:**: `toString` in interface `ChemRxn` **Specified by:**: `toString` in class `AbstractRxn`
    :   **Returns:**: an appropriate string representation of this reversible reaction.


---


|  |  |  |
| --- | --- | --- |
| **Overview** \| **Package** \| **Class** \| **Use** \| **Tree** \| **Deprecated** \| **Index** \| **Help** | |  |
| **PREV CLASS**   **NEXT CLASS** | **FRAMES**    **NO FRAMES**     **All Classes** |  |
| SUMMARY: NESTED \| FIELD \| CONSTR \| METHOD | DETAIL: FIELD \| CONSTR \| METHOD |  |


---
